# Children’s Continuous Medicaid Eligibility During COVID-19 and Health Care Access, Use, and Barriers to Care

**DOI:** 10.1001/jamahealthforum.2025.1376

**Published:** 2025-06-13

**Authors:** Erica L. Eliason, Daniel B. Nelson, Jordan Wood, Doug Strane, Aditi Vasan

**Affiliations:** 1 Center for State Health Policy, Rutgers University, New Brunswick, New Jersey; 2 Department of Urban-Global Public Health, Rutgers School of Public Health, Newark, New Jersey; 3 Division of General Internal Medicine and Geriatrics, Department of Medicine, School of Medicine, Oregon Health & Science University, Portland; 4 PolicyLab, Children’s Hospital of Philadelphia, Philadelphia; 5 Department of Pediatrics, University of Pennsylvania Perelman School of Medicine, Philadelphia

## Abstract

**Importance:**

National continuous Medicaid eligibility under the Families First Coronavirus Response Act (FFCRA) may have differentially affected children’s health care depending on whether states had preexisting 12-month continuous Medicaid eligibility for children.

**Objective:**

To estimate the association of states newly implementing continuous Medicaid eligibility under the FFCRA with children’s health care access, health care use, and barriers to care.

**Design, Setting, and Participants:**

This survey study used a difference-in-differences research design comparing states before (2017-2019) and during (2020-2022) the FFCRA overall, by caregiver-reported race and ethnicity, and among publicly insured children. Analyses used data from the National Survey of Children’s Health (NSCH), an annual household survey on the health and well-being of children 0 to 17 years old in the US. Data were analyzed from September 2024 to March 2025.

**Exposures:**

Whether states had pre-FFCRA 12-month continuous Medicaid eligibility for children.

**Main Outcomes and Measures:**

Insurance coverage, gaps in coverage, unmet health care needs, any health care visits, preventive visits, emergency department visits, hospitalizations, any time spent weekly arranging children’s health care, and problems paying medical bills.

**Results:**

The sample included 215 884 children, with children in states with pre-FFCRA continuous eligibility being similar to children in states newly implementing continuous eligibility with respect to age (8.6 years old in both sets of states), gender (49.6% female compared to 48.5%), and nativity (66.7% third generation or longer with all parents born in the US vs 69.6%), with lower proportions who were non-Hispanic Black (11.9% compared to 13.8%) or non-Hispanic White (50.5% compared to 52.9%), and higher proportions who were Hispanic (25.5% compared to 23.9%). In adjusted difference-in-difference models, newly implementing continuous eligibility under the FFCRA was associated with a 0.7–percentage point (95% CI, −1.2 to −0.1 percentage point) reduction in children’s unmet health care needs. There was no evidence of additional FFCRA-associated changes in outcomes overall. In subgroup analyses, there were reductions in coverage gaps, unmet health care needs, and time spent arranging care among Hispanic children and publicly insured children.

**Conclusions and Relevance:**

In this survey study, newly implementing continuous eligibility for children under the FFCRA was associated with reductions in unmet health care needs and no additional changes in health care outcomes overall, with additional benefits for Hispanic children and publicly insured children. This could reflect expected changes under mandatory, national 12-month continuous eligibility for children implemented in January 2024.

## Key Points

**Question:**

What was the association of states newly implementing continuous Medicaid eligibility under the Families First Coronavirus Response Act (FFCRA) with children’s health care access, health care use, and barriers to care?

**Findings:**

In this survey study of 215 884 children, newly implementing continuous Medicaid eligibility under the FFCRA was associated with reductions in children’s unmet care needs overall compared to states with preexisting continuous eligibility, with additional benefits among Hispanic and publicly insured children.

**Meaning:**

Continuous Medicaid eligibility under the FFCRA was associated with declines in children’s unmet care needs, with implications for mandatory, national 12-month continuous eligibility for children implemented in January 2024.

## Teaser

This survey study estimates the association of states newly implementing continuous Medicaid eligibility under the Families First Coronavirus Response Act with children’s health care access, health care use, and barriers to care.

## Introduction

Policies that support continuous enrollment in health insurance coverage for children have been found to increase coverage duration, decrease unmet health care needs, and improve health outcomes.^1,2^ Even short periods of uninsurance among children have been associated with a lower likelihood of having a usual source of care and a higher likelihood of delays in necessary care and unmet health care needs.^3,4,5,6^ Since 1997, states have had the option to offer 12 months of continuous Medicaid eligibility for children, allowing children to retain Medicaid for 1 year regardless of fluctuations in household income that may have otherwise affected their eligibility.^7^ By 2011, 23 states had adopted this policy, with 24 states offering 12-month continuous Medicaid eligibility for children by 2017 to 2019.^8,9,10^

During the COVID-19 public health emergency, the March 2020 Families First Coronavirus Response Act (FFCRA) promoted access to care by offering states enhanced federal funding in exchange for enacting continuous Medicaid eligibility.^11^ As all states adopted this policy, all Medicaid beneficiaries retained coverage during the FFCRA unless individuals actively disenrolled or moved outside of the state.^11^ When implemented, FFCRA continuous eligibility was indefinite, originally through the end of the public health emergency.^12^ Research has found that FFCRA continuous eligibility was associated with increased public coverage continuity and reduced uninsurance for children,^13,14^ with larger coverage gains in states that did not have prior 12-month continuous eligibility policies for children and, therefore, newly implemented continuous eligibility.^15,16^ However, to our knowledge, no studies have examined whether implementing continuous eligibility under the FFCRA was associated with changes in children’s health care outcomes. This research could have immediate relevance, as 12-month continuous eligibility became mandatory in all states starting in January 2024, under the Consolidated Appropriations Act (CAA) of 2023, and 13 states are implementing or pursuing multiyear continuous eligibility for young children, which may more closely mimic children’s coverage patterns during the FFCRA.^17,18^ Thus, the objective of this study was to estimate the association of states newly implementing continuous Medicaid eligibility under the FFCRA with children’s access to health services, health care use, and barriers to care.

## Methods

### Data and Sample

We used data from the 2017-2022 National Survey of Children’s Health (NSCH), an annual household survey on the health and well-being of children 0 to 17 years old conducted by the US Census Bureau on behalf of the Maternal and Child Health Bureau of the Health Resources and Services Administration (eTable 1 in Supplement 1).^19^ This study was determined to be nonhuman participant research by the Rutgers University institutional review board. We followed the Strengthening the Reporting of Observational Studies in Epidemiology (STROBE) reporting guidelines.

### Measures

Outcomes included (1) insurance at the time of the interview (public coverage, private coverage, or uninsurance), (2) gaps in health coverage, (3) unmet health care needs, (4) any health care visits, (5) preventive care visits, (6) emergency department visits, (7) hospitalizations, (8) any time spent arranging or coordinating health care for the child (eg, making appointments, locating services), and (9) problems paying health care bills. All outcomes were measured with a reference period of the previous 12 months, except for current insurance coverage and spending any time arranging health care for the child, which was asked in reference to an average week. All outcomes were asked for the full 2017-2022 study period except for hospitalizations, which were not included in the 2017 survey.

We considered demographic characteristics, including the child’s age, gender, and caregiver-reported race and ethnicity (Hispanic, non-Hispanic Asian, non-Hispanic Black, non-Hispanic Indigenous, non-Hispanic Native Hawaiian or Pacific Islander, non-Hispanic White, and non-Hispanic multiracial or other unlisted race); maternal age; highest parental educational attainment (high school or less or more than high school); nativity (first-generation household with child and parent born outside of the US, second-generation household with ≥1 parent born outside of the US, third-generation and longer with all parents born in the US, or other [ie, child born in US but parents not listed]); household number of children; and household language (English, Spanish, or other). We included variables for race and ethnicity due to long-standing racism, discrimination, and inequities in children’s health care access in the US.^20^

### Study Design

We used a difference-in-differences research design to estimate the association of newly implementing continuous Medicaid eligibility for children with health care outcomes before (2017-2019) and during (2020-2022) national continuous eligibility under the FFCRA. By comparing the 26 states and Washington, DC, newly implementing continuous Medicaid eligibility for children under the FFCRA to the 24 states with preexisting 12-month continuous eligibility policies (eTable 2 in Supplement 1), this approach controls for secular trends affecting children’s health care outcomes, including changes due to the COVID-19 pandemic. We estimated models overall; by race and ethnicity for Hispanic, non-Hispanic Black, and non-Hispanic White children; and among children who reported current public insurance, defined as Medicaid, medical assistance, or any kind of government insurance plan for those with low incomes or a disability. We were not powered for analyses among further racial and ethnic groups.

### Statistical Analysis

We estimated linear probability models for each outcome. The primary exposure variable was an interaction term between pre-FFCRA state continuous eligibility status (ie, whether a state had a pre-FFCRA 12-month continuous eligibility policy for children) and time period (whether the survey year was before or during the FFCRA). We included state and year fixed effects for all models, with covariates at the child, parent, and household level. We calculated heteroskedasticity-robust standard errors clustered by state, the level of policy variation, to account for correlation of error terms at the state level. We used NSCH sample weights to adjust for the probability of selection and nonresponse in the survey, and to be representative of noninstitutionalized children 0 to 17 years old at the state and national levels.^21^ Annual survey weights were combined according to the NSCH guide for multiyear analyses.^22^ Stata, version 18.5 (StataCorp), was used in analyses. A 2-sided *P* < .05 was considered statistically significant.

### Supplemental Analysis

We conducted several analyses to examine the parallel trends assumption for the difference-in-differences research design, which presumes that trends in the outcomes would not have changed differentially between the treated group (states newly implementing continuous eligibility for children) and control group (states with preexisting 12-month continuous eligibility) in the absence of the intervention (the FFCRA). We assessed whether trends were diverging by state continuous eligibility status prior to the FFCRA by testing for linear differences in the pre-FFCRA trends, estimating pre-FFCRA event study coefficients, and plotting the unadjusted trends in the outcomes (eTables 3 and 4 and the eFigure in Supplement 1). As continuous eligibility could affect the demographic composition of publicly insured children, we examined whether FFCRA continuous eligibility was associated with differential changes in demographic characteristics among children with public insurance by pre-FFCRA continuous eligibility status (eTable 5 in Supplement 1). As 2020 was a transitional period, we estimated separate models omitting 2020 (eTable 6 in Supplement 1). To confirm that no particular states were driving the results, we estimated 5 models omitting the states with the highest numbers of respondent children in the survey (eTable 7 in Supplement 1). Finally, as a sensitivity analysis, we included federal poverty level as an additional covariate in the models, accounting for the multiple imputation framework for this variable (eTable 8 in Supplement 1).^21,23^ Data were analyzed from September 2024 to March 2025.

## Results

The sample included a total of 215 884 respondents, representing a weighted total of 67 694 839 children. Table 1 summarizes demographic characteristics by pre-FFCRA continuous eligibility status. Prior to the FFCRA, children residing in the 24 states with 12-month continuous eligibility, compared to children in the 26 states and Washington, DC, without 12-month continuous eligibility, were comparable in age (mean [SD], 8.6 [5.2] years compared to 8.6 [5.1] years) and gender (49.6% female compared to 48.5%). There were lower proportions of children in states with existing 12-month continuous eligibility compare to without who were non-Hispanic Black (11.9% compared to 13.8%) or non-Hispanic White (50.5% compared to 52.9%), and higher proportions who were Hispanic (25.5% compared to 23.9%). Children’s parents in states with and without pre-FFCRA continuous eligibility had comparable levels of educational attainment (73.3% had more than high school education attainment compared to 72.1%) and maternal age (mean [SD], 29.7 [6.0] years vs 29.3 [6.0] years). Similar proportions of children in states with continuous eligibility compared to without had household nativity of third generation or more (66.7% compared to 69.6%) and comparable household number of children (39.4% with 2 children compared to 38.2%) and household language used (English among 85.6% compared to 86.2%).

**Table 1.** Demographic Characteristics Among the Sample by State Continuous Eligibility (CE) Adoption Status, 2017-2019^a^

Characteristic	%^b^	
	States with existing 12-mo CE (n = 36 250)	States newly implementing CE (n = 40 790)
Child age, mean (SD), y	8.6 (5.2)	8.6 (5.1)
Child gender		
Female	49.6	48.5
Male	50.4	51.5
Child race and ethnicity^c^		
Hispanic	25.5	23.9
Non-Hispanic Asian	5.6	3.6
Non-Hispanic Black	11.9	13.8
Non-Hispanic Indigenous	0.3	0.4
Non-Hispanic Native Hawaiian or Pacific Islander	0.1	0.2
Non-Hispanic White	50.5	52.9
Non-Hispanic multiracial or other race	6.0	5.3
Age of mother, mean (SD), y	29.7 (6.0)	29.3 (6.0)
Parental education		
High school or less	26.7	27.9
More than high school	73.3	72.1
Household nativity		
First generation	2.6	2.9
Second generation	25.2	21.5
Third generation or more	66.7	69.6
Other^d^	4.7	5.4
No. of children in household		
1	25.7	25.5
2	39.4	38.2
3	22.9	23.3
≥4	11.9	13.1
Household language used		
English	85.6	86.2
Spanish	9.3	9.6
Other language	5.1	4.2

^a^ Data are from the National Survey of Children’s Health. State continuous eligibility adoption status is determined by whether a state had existing 12-month continuous Medicaid eligibility policies for children from 2017 to 2019. Data are weighted using National Survey of Children’s Health survey weights.

^b^ Twenty-four states offered 12-month continuous Medicaid eligibility for children, and 26 states and Washington, DC, were newly implementing.

^c^ Race and ethnicity were reported by the caregiver. Non-Hispanic multiracial or other race includes those reporting a race that is unlisted in the National Survey of Children’s Health.

^d^ Child was born in US but parents not listed.

In adjusted difference-in-difference models, newly implementing continuous eligibility for children under the FFCRA was associated with a 0.7–percentage point (pp; 95% CI, −1.2 to −0.1) reduction in unmet health care needs in the past year, or a 21% decrease from baseline levels, relative to states with preexisting 12-month continuous eligibility (Table 2). Among children overall, there was no evidence of an association between newly implementing continuous eligibility and children’s current insurance, coverage gaps, health care visits, preventive checkups, emergency department visits, hospital stays, or problems paying for health care.

**Table 2.** Difference-in-Differences Estimates of the Association of State Continuous Eligibility (CE) Adoption Status for Children and Health Care Access, Use, and Barriers Overall, 2017-2022^a^

Outcome	States with existing 12-mo CE, %^b^		Difference (95% CI), pp	States newly implementing CE, %^b^		Difference (95% CI), pp	Difference-in-differences (95% CI)	
	Pre-FFCRA (n = 36 250)	During FFCRA (n = 71 936)		Pre-FFCRA (n = 40 790)	During FFCRA (n = 66 908)		Unadjusted	Adjusted
Current public coverage	35.6	35.8	0.2 (−1.3 to 1.7)	34.4	34.5	0.1 (−1.3 to 1.5)	−0.2 (−1.5 to 1.1)	0.5 (−0.5 to 1.4)
Current private coverage	65.1	65.0	−0.1 (−1.6 to 1.4)	65.6	65.7	0.1 (−1.3 to 1.4)	0.2 (−1.0 to 1.4)	−0.4 (−1.7 to 0.9)
Current uninsurance	4.8	5.2	0.4 (−0.3 to 1.1)	7.7	7.7	0.1 (−0.9 to 1.0)	−0.4 (−1.1 to 0.4)	−0.2 (−1.0 to 0.5)
Gaps in health coverage	6.7	6.6	−0.2 (−1.0 to 0.6)	10.1	9.0	−1.1 (−2.1 to −0.1)	−0.9 (−2.1 to 0.2)	−0.8 (−1.9 to 0.3)
Unmet health care needs	2.7	3.6	0.9 (0.4 to 1.4)	3.3	3.6	0.3 (−0.3 to 0.9)	−0.7 (−1.2 to −0.1)	−0.7 (−1.2 to −0.1)
Any health care visit	82.8	81.9	−0.9 (−2.1 to 0.3)	82.7	82.2	−0.5 (−1.6 to 0.6)	0.4 (−1.5 to 2.3)	0.2 (−1.6 to 2.0)
Preventive checkup visit	79.6	77.9	−1.7 (−2.9 to −0.4)	79.3	78.4	−0.9 (−2.1 to 0.2)	0.8 (−1.5 to 3.0)	0.5 (−1.6 to 2.7)
Emergency department visit	19.7	14.7	−5.0 (−6.1 to −3.9)	19.4	15.8	−3.6 (−4.6 to −2.5)	1.4 (−0.3 to 3.2)	1.5 (−0.2 to 3.2)
Hospital stay	3.9	2.9	−0.9 (−1.6 to −0.3)	3.8	3.0	−0.7 (−1.2 to −0.2)	0.2 (−0.3 to 0.8)	0.3 (−0.3 to 0.8)
Spent time arranging child’s health care	10.5	10.1	−0.4 (−1.2 to 0.4)	11.9	10.4	−1.5 (−2.3 to −0.7)	−1.1 (−2.8 to 0.5)	−1.1 (−2.8 to 0.5)
Problems paying health care bills	9.4	7.6	−1.7 (−2.5 to −1.0)	11.6	9.4	−2.2 (−3.0 to −1.3)	−0.4 (−1.7 to 0.9)	−0.6 (−1.8 to 0.7)

Abbreviations: FFCRA, Families First Coronavirus Response Act; pp, percentage point.

^a^ Data are from the National Survey of Children’s Health. State continuous eligibility adoption status is determined by whether a state had existing 12-month continuous Medicaid eligibility policies for children from 2017 to 2019. Hospitalization data were only available from 2018 to 2022. Data are weighted using National Survey of Children’s Health survey weights.

^b^ Twenty-four states offered 12-month continuous Medicaid eligibility for children, and 26 states and Washington, DC, were newly implementing.

In adjusted models stratified by caregiver-reported race and ethnicity, there was no evidence of an association between newly implementing continuous eligibility for children under the FFCRA and any health care outcomes among non-Hispanic Black or non-Hispanic White children (Table 3). Among Hispanic children, newly implementing continuous eligibility under the FFCRA was associated with a 2.8-pp (95% CI, 0.7-5.0 pp) increase in current public coverage, a 3.3-pp (95% CI, −5.1 to −1.6 pp) decrease in coverage gaps, and a 1.5-pp (95% CI, −2.5 to −0.5 pp) decrease in unmet health care needs in the past year. Hispanic children also experienced a 2.9-pp (95% CI, 0.2-5.6 pp) increase in preventive checkups in the past year and a 3.5-pp (95% CI, −5.5 to −1.5 pp) decrease in their families spending any time coordinating the child’s health care in an average week associated with newly implementing continuous eligibility. Among Hispanic children, there was no evidence of an association between newly implementing continuous eligibility under the FFCRA and current private coverage, current uninsurance, overall health care visits, emergency department visits, hospital stays, or problems paying health care bills.

**Table 3.** Difference-in-Differences Estimates of the Association of State Continuous Eligibility (CE) Adoption Status for Children and Health Care Access, Use, and Barriers by Race and Ethnicity, 2017-2022^a^

Outcome	States with existing 12-mo CE, %^b^		Difference (95% CI), pp	States newly implementing CE, %^b^		Difference (95% CI), pp	Difference-in-differences (95% CI)	
	Pre-FFCRA	During FFCRA		Pre-FFCRA	During FFCRA		Unadjusted	Adjusted
**Hispanic children (n = 28 141)**								
Current public coverage	52.0	51.9	−0.1 (−4.2 to 4.1)	50.4	50.2	−0.2 (−3.9 to 3.6)	−0.0 (−2.3 to 2.3)	2.8 (0.7 to 5.0)
Current private coverage	48.5	48.6	0.2 (−4.0 to 4.3)	48.9	49.8	1.0 (−2.7 to 4.7)	0.7 (−1.9 to 3.3)	−2.0 (−4.6 to 0.6)
Current uninsurance	6.1	7.4	1.2 (−0.8 to 3.3)	13.3	12.6	−0.7 (−3.6 to 2.2)	−1.8 (−3.2 to −0.4)	−1.2 (−2.6 to 0.2)
Gaps in health coverage	8.7	9.7	1.1 (−1.4 to 3.5)	17.9	14.9	−3.1 (−6.2 to 0.1)	−4.0 (−5.6 to −2.4)	−3.3 (−5.1 to −1.6)
Unmet health care needs	3.5	4.7	1.3 (−0.2 to 2.8)	4.6	4.2	−0.4 (−2.1 to 1.4)	−1.7 (−2.7 to −0.6)	−1.5 (−2.5 to −0.5)
Any health care visit	77.4	75.7	−1.7 (−5.2 to 1.8)	75.0	76.2	1.2 (−2.1 to 4.5)	2.8 (0.0 to 5.6)	1.8 (−1.1 to 4.6)
Preventive checkup visit	74.3	71.1	−3.3 (−7.0 to 0.4)	71.8	72.6	0.8 (−2.6 to 4.1)	4.0 (1.3 to 6.7)	2.9 (0.2 to 5.6)
Emergency department visit	4.4	3.1	−1.3 (−3.2 to 0.5)	3.5	3.2	−0.3 (−1.7 to 1.0)	1.0 (−0.0 to 2.1)	1.0 (−0.1 to 2.0)
Hospital stay	21.3	15.3	−6.0 (−9.1 to −2.8)	21.2	17.2	−4.0 (−6.9 to −1.1)	2.1 (−0.4 to 4.5)	1.8 (−0.4 to 4.1)
Spent time arranging child’s health care	8.7	9.7	1.0 (−1.2 to 3.1)	11.7	9.3	−2.4 (−4.5 to −0.3)	−3.3 (−5.4 to −1.2)	−3.5 (−5.5 to −1.5)
Problems paying health care bills	8.4	8.2	−0.2 (−2.2 to 1.7)	12.0	10.7	−1.3 (−3.8 to 1.2)	−1.0 (−3.5 to 1.5)	−1.5 (−3.9 to 0.9)
**Non-Hispanic Black children (n = 13 114)**								
Current public coverage	61.2	60.0	−1.2 (−5.5 to 3.2)	57.2	52.6	−4.6 (−8.9 to −0.3)	−3.4 (−7.0 to 0.2)	−2.5 (−5.3 to 0.4)
Current private coverage	42.0	43.6	1.6 (−2.8 to 6.0)	46.7	48.3	1.7 (−2.7 to 6.0)	0.1 (−5.2 to 5.3)	−1.0 (−4.7 to 2.8)
Current uninsurance	5.8	6.1	0.3 (−1.8 to 2.3)	7.3	7.7	0.4 (−2.1 to 2.9)	0.1 (−3.0 to 3.2)	−0.1 (−3.2 to 2.9)
Gaps in health coverage	9.5	8.3	−1.2 (−3.7 to 1.3)	10.6	9.5	−1.1 (−3.9 to 1.7)	0.2 (−3.8 to 4.2)	−0.1 (−3.7 to 3.6)
Unmet health care needs	3.7	4.7	0.9 (−1.0 to 2.9)	4.7	3.3	−1.4 (−3.8 to 0.9)	−2.3 (−5.3 to 0.8)	−2.4 (−5.2 to 0.5)
Any health care visit	79.3	80.4	1.1 (−2.4 to 4.6)	82.8	80.0	−2.8 (−6.1 to 0.5)	−4.1 (−8.4 to 0.3)	−4.0 (−8.4 to 0.4)
Preventive checkup visit	76.2	76.8	0.6 (−3.1 to 4.2)	80.0	76.8	−3.2 (−6.7 to 0.3)	−3.9 (−8.8 to 1.0)	−3.9 (−8.9 to 1.0)
Emergency department visit	3.9	3.9	0.0 (−1.6 to 1.7)	4.9	3.6	−1.3 (−3.1 to 0.5)	−1.3 (−3.4 to 0.9)	−1.1 (−3.2 to 1.0)
Hospital stay	29.9	21.3	−8.7 (−12.5 to −4.8)	29.4	22.0	−7.4 (−11.2 to −3.5)	1.3 (−5.2 to 7.9)	1.6 (−4.3 to 7.5)
Spent time arranging child’s health care	13.3	8.9	−4.3 (−6.8 to −1.8)	14.4	11.0	−3.4 (−6.3 to −0.5)	0.9 (−2.9 to 4.7)	0.7 (−3.1 to 4.5)
Problems paying health care bills	9.3	7.1	−2.2 (−4.5 to 0.1)	12.8	8.9	−3.9 (−7.0 to −0.9)	−1.8 (−4.2 to 0.7)	−2.0 (−4.5 to 0.6)
**Non-Hispanic White children (n = 145 870)**								
Current public coverage	23.1	24.1	1.0 (−0.3 to 2.4)	22.3	23.3	1.0 (−0.3 to 2.3)	−0.0 (−1.6 to 1.6)	0.0 (−1.3 to 1.4)
Current private coverage	77.1	76.5	−0.6 (−1.9 to 0.7)	76.9	76.6	−0.3 (−1.6 to 1.0)	0.2 (−1.4 to 1.8)	0.1 (−1.5 to 1.7)
Current uninsurance	4.2	3.9	−0.3 (−1.0 to 0.4)	5.5	5.7	0.3 (−0.5 to 1.0)	0.5 (−0.3 to 1.4)	0.6 (−0.2 to 1.4)
Gaps in health coverage	5.4	4.5	−0.9 (−1.7 to −0.2)	6.8	6.4	−0.4 (−1.2 to 0.4)	0.5 (−0.6 to 1.6)	0.5 (−0.5 to 1.6)
Unmet health care needs	2.2	2.9	0.7 (0.3 to 1.2)	2.5	3.4	0.9 (0.4 to 1.4)	0.2 (−0.7 to 1.0)	0.2 (−0.7 to 1.0)
Any health care visit	86.7	86.5	−0.2 (−1.3 to 0.9)	86.5	85.9	−0.6 (−1.6 to 0.4)	−0.4 (−1.7 to 1.0)	−0.4 (−1.7 to 0.9)
Preventive checkup visit	83.3	82.7	−0.5 (−1.7 to 0.6)	82.8	81.9	−1.0 (−2.1 to 0.2)	−0.4 (−1.8 to 1.1)	−0.4 (−1.8 to 1.0)
Emergency department visit	3.8	2.7	−1.1 (−1.7 to −0.5)	3.5	2.8	−0.7 (−1.3 to −0.2)	0.3 (−0.3 to 1.0)	0.4 (−0.4 to 1.1)
Hospital stay	16.8	13.5	−3.3 (−4.4 to −2.2)	16.6	13.9	−2.7 (−3.7 to −1.7)	0.6 (−1.0 to 2.2)	0.6 (−1.0 to 2.2)
Spent time arranging child’s health care	10.9	10.6	−0.3 (−1.1 to 0.6)	11.3	10.8	−0.5 (−1.3 to 0.4)	−0.2 (−1.4 to 1.0)	−0.1 (−1.3 to 1.0)
Problems paying health care bills	10.3	7.8	−2.5 (−3.3 to −1.7)	11.3	9.3	−2.1 (−2.9 to −1.2)	0.4 (−0.7 to 1.6)	0.4 (−0.8 to 1.7)

Abbreviations: FFCRA, Families First Coronavirus Response Act; pp, percentage point.

^a^ Data are from the National Survey of Children’s Health. State continuous eligibility adoption status is determined by whether a state had existing 12-month continuous Medicaid eligibility policies for children from 2017 to 2019. Hospitalization data were only available from 2018 to 2022. Data are weighted using National Survey of Children’s Health survey weights. Race and ethnicity were reported by the caregiver. Analyses are only among Hispanic children, non-Hispanic Black children, and non-Hispanic White children because the study was not powered for analyses among further racial and ethnic groups.

^b^ Twenty-four states offered 12-month continuous Medicaid eligibility for children, and 26 states and Washington, DC, were newly implementing.

Among publicly insured children, newly implementing continuous eligibility under the FFCRA was associated with reductions in coverage gaps in the past year by 2.2 pp (95% CI, −3.9 to −0.4 pp), reductions in unmet health care needs in the past year by 1.4 pp (95% CI, −2.7 to 0.0 pp), and reductions in spending any time coordinating children’s health care in an average week by 2.5 pp (95% CI, −5.0 to 0.0 pp) (Table 4). There was no evidence of an association between newly implementing continuous eligibility under the FFCRA and health care visit outcomes or problems paying health care bills among children with public insurance.

**Table 4.** Difference-in-Differences Estimates of the Association of State Continuous Eligibility (CE) Adoption Status for Children and Health Care Access, Use, and Barriers Among Publicly Insured Children, 2017-2022^a^

Outcome	States with existing 12-mo CE, %^b^		Difference (95% CI), pp	States newly implementing CE, %^b^		Difference (95% CI), pp	Difference-in-differences (95% CI)	
	Pre-FFCRA (n = 8700)	During FFCRA (n = 19 114)		Pre-FFCRA (n = 8744)	During FFCRA (n = 15 689)		Unadjusted	Adjusted
Gaps in health coverage	3.6	2.7	−1.0 (−2.0 to 0.1)	5.7	2.6	−3.2 (−4.4 to −1.9)	−2.2 (−4.0 to −0.4)	−2.2 (−3.9 to −0.4)
Unmet health care needs	3.6	5.3	1.7 (0.6 to 2.8)	4.4	4.8	0.4 (−0.9 to 1.7)	−1.3 (−2.6 to 0.0)	−1.4 (−2.7 to −0.0)
Any health care visit	78.5	79.9	1.4 (−1.1 to 3.8)	82.3	82.6	0.4 (−1.7 to 2.5)	−1.2 (−3.5 to 1.2)	−1.5 (−3.9 to 0.9)
Preventive checkup visit	75.4	74.9	−0.5 (−3.1 to 2.1)	79.0	78.3	−0.8 (−3.0 to 1.5)	−0.4 (−2.6 to 1.9)	−0.6 (−2.7 to 1.4)
Emergency department visit	5.4	3.9	−1.5 (−2.9 to −0.1)	5.7	4.5	−1.3 (−2.6 to 0.1)	0.3 (−1.2 to 1.8)	0.3 (−1.2 to 1.8)
Hospital stay	28.1	20.6	−7.5 (−10.0 to −5.0)	30.1	23.7	−6.4 (−8.9 to −3.9)	1.3 (−1.0 to 3.5)	1.5 (−0.6 to 3.6)
Spent time arranging child’s health care	13.1	12.2	−0.8 (−2.6 to 0.9)	17.1	13.9	−3.3 (−5.1 to −1.4)	−2.3 (−4.9 to 0.2)	−2.5 (−5.0 to −0.0)
Problems paying health care bills	5.3	5.1	−0.2 (−1.2 to 0.8)	7.2	6.2	−1.0 (−2.7 to 0.6)	−0.8 (−2.3 to 0.7)	−1.1 (−2.6 to 0.5)

Abbreviations: FFCRA, Families First Coronavirus Response Act; pp, percentage point.

^a^ Data are from the National Survey of Children’s Health. State continuous eligibility adoption status is determined by whether a state had existing 12-month continuous Medicaid eligibility policies for children from 2017 to 2019. Hospitalization data were only available from 2018 to 2022. Data are weighted using National Survey of Children’s Health survey weights.

^b^ Twenty-four states offered 12-month continuous Medicaid eligibility for children, and 26 states and Washington, DC, were newly implementing.

In supplemental analyses, there was no evidence of differential changes in demographic characteristics among publicly insured children during the FFCRA by state continuous eligibility status, suggesting that changes in demographic composition were not associated with changes in health care outcomes during the FFCRA (eTable 5 in Supplement 1). In analyses omitting 2020 as a transition year, in the 5 models omitting states with the highest numbers of children in the survey, and in models using a multiple imputation approach to control for federal poverty level as an additional covariate, results were similar to main models regarding effect size and statistical significance for most outcomes (eTables 6-8 in Supplement 1). In models omitting 2020 as a transition year, newly implementing continuous eligibility under the FFCRA was additionally associated with a 2.1-pp (95% CI, 0.4-3.7 pp) increase in children’s emergency department visits relative to states with preexisting continuous eligibility (eTable 6 in Supplement 1).

## Discussion

In this difference-in-differences analysis examining changes in children’s access to health services, health care use, and barriers to care during national continuous Medicaid eligibility under the FFCRA, we found an association between new state-level implementation of continuous eligibility for children and reductions in children’s unmet health care needs. Among Hispanic children and publicly insured children, there were additional benefits associated with newly implementing continuous eligibility, including improvements in coverage stability and reductions in the time families spent arranging and coordinating children’s health care services. Among Hispanic children only, newly implementing continuous eligibility was also associated with an increase in preventive care receipt. We found no evidence among any group of changes to children’s overall health care visits, hospital stays, emergency department use, or problems paying for care.

While prior studies using Medicaid administrative data found larger gains in children’s Medicaid enrollment in states newly implementing continuous eligibility under the FFCRA compared to states with prior 12-month continuous eligibility, to our knowledge, this study is the first to examine whether newly implementing continuous eligibility was associated with caregiver-reported changes in children’s health care access, health care use, and barriers to care.^15,16^ Our use of caregiver-reported data to measure children’s access to care is especially important, as recent analyses of survey data have found that many Medicaid enrollees may have been unaware of their continuous coverage and mistakenly believed that they were uninsured during the FFCRA (the “Medicaid undercount”).^24,25^ Our stratified analyses restricted to the sample of children reporting public coverage therefore likely reflect changes among children whose families were aware of their public coverage enrollment, and who would have been more able to benefit from continuous eligibility.

Although we observed a reduction in children’s unmet health care needs, we did not observe substantial changes in children’s health care use overall. This study measured only whether children received each included type of health care visit in the past year, but not the number of health care visits in each setting. As we found reductions in children’s unmet care needs, it is possible that continuous eligibility may have had an impact on the frequency of health care visits, which we were not able to measure in these data. These findings could also potentially indicate changes in access to telehealth, prescription medications, or other services for children outside of the definition of health care visits. Previous research found that the COVID-19 pandemic was associated with disruptions in children’s health care use, with meaningful decreases in preventive care, specialist care, emergency department visits, hospitalizations, and pediatric well-child visits, including among publicly insured children.^26,27,28,29,30^ It is possible that continuous Medicaid eligibility policies could result in greater improvements in children’s health care use in a nonpandemic context.

States could begin rolling back continuous eligibility under the FFCRA unwinding in April 2023, resuming Medicaid disenrollment procedures over the next 12 to 14 months.^31^ Recent analyses suggest that up to 5.53 million children may have been disenrolled and lost Medicaid coverage as a result of the FFCRA unwinding.^32^ Children in states without prior 12-month continuous eligibility saw larger Medicaid gains under the FFCRA,^15,16^ which could have put them at greater risk of losing coverage during the unwinding.^33^ Beginning in January 2024, 12-month continuous Medicaid eligibility became mandatory for children in all states under the CAA of 2023.^17^ This work suggests that national 12-month continuous eligibility for children may help address unmet health care needs for children in states without preexisting continuous eligibility policies, with more than 17 million children potentially benefitting from the CAA.^34^ Consistent with prior research finding the largest reductions in uninsurance among Hispanic children during the FFCRA, the present results suggest that Hispanic children may particularly benefit from continuous eligibility policies.^35^ Improvements may be even greater in the 13 states that have recently implemented or are currently seeking approval to implement multiyear continuous eligibility for young children.^36^

### Limitations

This study had several limitations. First, implementation of continuous eligibility under the FFCRA coincided with the COVID-19 pandemic, which affected children’s health care outcomes.^26,27,28^ We controlled for the effects of the COVID-19 pandemic that affected all states by comparing states by pre-FFCRA continuous eligibility adoption to identify the association of newly implementing continuous eligibility for children with children’s health care outcomes. However, the findings of no association between newly implementing continuous eligibility and children’s health care use could potentially have been affected by the COVID-19 pandemic context, which may have dampened health care use among children in both sets of states. As a result, these findings may not be generalizable to other time periods and contexts, when continuous Medicaid eligibility for children could have greater implications for health care. Second, states with preexisting continuous Medicaid eligibility for children may be systematically different from states newly implementing these policies. However, we found no evidence to reject the parallel trends assumption, suggesting that the control states provide a plausible comparison for states newly implementing continuous eligibility. Third, our measures of health care use were limited to whether children received any health care in the past year. More detailed health care outcomes, such as the number of health care visits, may better capture changes in care associated with continuous eligibility. Fourth, this study relied on caregiver-reported outcomes with reference periods primarily referring to the previous 12 months, which may be subject to recall or reporting bias. Fifth, hospitalizations were not included in the 2017 NSCH, which limited our ability to establish parallel trends for this outcome due to fewer years of pre-FFCRA data. Finally, the association of continuous eligibility with children’s health care access, health care use, and barriers to care likely differed across characteristics, such as children’s nativity, household income, special health care needs, and further racial and ethnic groups. This study was limited by sample size for these subgroups, and we therefore could not examine how these associations may have differed across these characteristics. Future research should explore how continuous eligibility affected children’s health care outcomes across additional demographic groups and consider additional, more precise measures of health care use.

## Conclusions

This survey study found that children in states without prior continuous Medicaid eligibility policies experienced reductions in unmet health care needs overall during the FFCRA compared to children in states with previous 12-month continuous eligibility policies. Among Hispanic children and publicly insured children, there were additional declines in coverage gaps and families’ health care–related administrative burdens. We found no evidence of changes in overall health care visits, hospital stays, emergency department use, or problems paying for care for any children. There may be similar implications for children’s health care access with mandatory 12-month continuous Medicaid eligibility under the CAA, particularly for children in states implementing multiyear continuous eligibility, with the potential for larger effects on health care use in a nonpandemic context.

## Notes

Supplement 1. **eTable 1.** National Survey of Children’s Health Data, 2017-2022 **eTable 2.** States by Continuous Eligibility Adoption Status, 2017-2019 **eTable 3.** Difference-in-Difference Model and Assumptions Checks **eTable 4.** Event Study Pre-Period Coefficients **eFigure.** Unadjusted Trends by State Continuous Eligibility Adoption Status, 2017-2022 **eTable 5.** Differential Change in Demographic Characteristics of Publicly Insured Children by State Continuous Eligibility Adoption Status, 2017-2022 **eTable 6.** Difference-in-Difference Analysis Omitting 2020 as a Transition Year, 2017-2022 **eTable 7.** Difference-in-Difference Analysis Omitting States with the Highest Numbers of Respondents, 2017-2022 **eTable 8.** Difference-in-Difference Analysis with Multiply Imputed Federal Poverty Level, 2017-2022
eReferences

Supplement 2.
Data Sharing Statement
