# Supplementary material for: Children’s Continuous Medicaid Eligibility During COVID-19 and Health Care Access, Use, and Barriers to Care
Source: JAMA Health Forum. 2025 Jun 13;6(6):e251376. doi: 10.1001/jamahealthforum.2025.1376 (PMC12166482; doi:10.1001/jamahealthforum.2025.1376)
Supplement: Supplement 1. — eTable 1. National Survey of Children’s Health Data, 2017-2022 eTable 2. States by Continuous Eligibility Adoption Status, 2017-2019 eTable 3. Difference-in-Difference Model and Assumptions Checks eTable 4. Event Study Pre-Period Coefficients eFigure. Unadjusted Trends by State Continuous Eligibility Adoption Status, 2017-2022 eTable 5. Differential Change in Demographic Characteristics of Publicly Insured Children by State Continuous Eligibility Adoption Status, 2017-2022 eTable 6. Difference-in-Difference Analysis Omitting 2020 as a Transition Year, 2017-2022 eTable 7. Difference-in-Difference Analysis Omitting States with the Highest Numbers of Respondents, 2017-2022 eTable 8. Difference-in-Difference Analysis with Multiply Imputed Federal Poverty Level, 2017-2022 eReferences [file jamahealthforum-e251376-s001.pdf]

Supplemental Online Content

Eliason EL, Nelson DB, Wood J, Strane D, Vasan A. Children's continuous Medicaid eligibility during COVID-19 and health care access, use, and barriers to care. *JAMA Health Forum*. 2025;6(6):e251376. doi:10.1001/jamahealthforum.2025.1376

eTable 1. National Survey of Children's Health Data, 2017-2022
eTable 2. States by Continuous Eligibility Adoption Status, 2017-2019
eTable 3. Difference-in-Difference Model and Assumptions Checks
eTable 4. Event Study Pre-Period Coefficients
eFigure. Unadjusted Trends by State Continuous Eligibility Adoption Status, 2017-2022
eTable 5. Differential Change in Demographic Characteristics of Publicly Insured Children by State Continuous Eligibility Adoption Status, 2017-2022
eTable 6. Difference-in-Difference Analysis Omitting 2020 as a Transition Year, 2017-2022
eTable 7. Difference-in-Difference Analysis Omitting States with the Highest Numbers of Respondents, 2017-2022
eTable 8. Difference-in-Difference Analysis with Multiply Imputed Federal Poverty Level, 2017-2022
eReferences

This supplemental material has been provided by the authors to give readers additional information about their work.

© 2025 Eliason EL et al. *JAMA Health Forum*.

eTable 1. National Survey of Children's Health Data, 2017-2022

Below, we present the unweighted and weighted study sample sizes and the overall response range per state each year. Starting in 2020, the number of respondents increased (644 to 3,039 per state in 2020 compared to 474 to 651 in 2019), which was the product of a higher number of sampled households rather than a change in the response rate (HRSA, 2024). Evaluations of the NSCH data found no changes in sample composition comparing 2020 data to earlier years, and concluded that state and national estimates from 2020 NSCH data were representative and comparable to previous survey years (U.S. Census Bureau, 2024a). As shown below, weighting the NSCH survey data produces comparable annual weighted sample sizes for the included study years to be representative of the US population of non-institutionalized children ages 0-17.

Year	Overall State Survey Response Range	Unweighted Study Sample	Weighted Study Sample
2017	343 to 470	20,342	11306347.6
2018	520 to 769	28,966	11393681.7
2019	474 to 651	27,732	11264353.2
2020	644 to 3,039	40,322	11277006.1
2021	788 to 2,956	47,807	11187779.4
2022	688 to 4,724	50,715	11265671.4
		Total: 215,884	Total: 67,694,839

© 2025 Eliason EL et al. *JAMA Health Forum*.

eTable 2. States by Continuous Eligibility Adoption Status, 2017-2019

States with Existing 12-Month CE (24 States)	States Newly Implementing CE (26 States and DC)
Alabama	Arizona
Alaska	Arkansas
California	Connecticut
Colorado	Delaware
Idaho	District of Columbia
Illinois	Florida
Iowa	Georgia
Kansas	Hawaii
Louisiana	Indiana
Maine	Kentucky
Michigan	Maryland
Mississippi	Massachusetts
Montana	Minnesota
New Jersey	Missouri
New Mexico	Nebraska
New York	Nevada
North Carolina	New Hampshire
North Dakota	Oklahoma
Ohio	Pennsylvania
Oregon	Rhode Island
South Carolina	South Dakota
Washington	Tennessee
West Virginia	Texas
Wyoming	Utah
	Vermont
	Virginia
	Wisconsin

Notes: State continuous eligibility adoption status is determined by whether a state had existing 12-month Medicaid continuous eligibility policies for children prior to implementation of continuous eligibility under the FFCRA. CE is continuous eligibility.

© 2025 Eliason EL et al. *JAMA Health Forum*.

eTable 3. Difference-in-Difference Model and Assumptions Checks

Difference-in-difference model specification:

$$\Upsilon_{IST} = \beta_0 + \beta_1(FFCRA * NewCE)_{IST} + \beta_2 YearFE_T + \beta_3 StateFE_{IS} + \chi_{IST} + \epsilon_{IST}$$

Our outcomes are Υ_{IST}, capturing children's health care access, use, and barriers to care. $\beta_1(FFCRA * NewCE)_{IST}$ represents our primary coefficient of interest, the difference-in-difference, measuring changes in states newly implementing continuous eligibility (CE) in Medicaid during the Families First Coronavirus Response Act (FFCRA) relative to states with prior 12-month continuous eligibility policies for children. All models include state and year fixed effects, $\beta_2 YearFE_T + \beta_3 StateFE_{IS}$, to control for time-invariant and state-invariant differences in the states and years across survey years and states. χ_{IST} is vector of control variables, including the child's age in years, gender (female, male), race and ethnicity (Non-Hispanic Asian, non-Hispanic Black, Hispanic, Non-Hispanic Indigenous, Non-Hispanic Native Hawaiian or Pacific Islander, non-Hispanic White, non-Hispanic other race), age of the mother in years, highest parental educational attainment (high school or less, more than high school), nativity (1st generation household with child and parent born outside the U.S., 2nd generation household with child or at least one parent born outside the U.S., 3rd+ generation with all parents born in the U.S., other), household number of children (1, 2, 3, 4 or more), and household language (English, Spanish, other language).

We conducted several analyses to examine the parallel trends assumption for the difference-in-difference research design, which presumes that trends in the outcomes would not have changed differentially between treated (states newly implementing continuous eligibility for children) and control (states with existing 12-month continuous eligibility policies) groups in the absence of the intervention (the FFCRA). We assessed whether trends diverged by state continuous eligibility adoption status prior to the FFCRA by plotting the unadjusted trends of the outcomes, testing for linear trend differences in the pre-policy period (below), and estimating pre-policy event study coefficients. To test for linear differences in the pre-FFCRA trends by state continuous eligibility adoption status, we interacted treated and control group status with a linear time trend with the state and year fixed effects and control variables presented in the primary difference-in-difference model:

$$\Upsilon_{IST} = \beta_0 + \beta_1(LinearTime * NewCE)_{IST} + \beta_2 YearFE_T + \beta_3 StateFE_{IS} + \chi_{IST} + \epsilon_{IST}$$

$\beta_1(LinearTime * NewCE)_{IST}$, the pre-FFCRA differential trend, captures the difference in pre-FFCRA trends in the outcomes by state continuous eligibility adoption status. We found no evidence of significant differential trends in the pre-FFCRA period among treated and control group states:

Outcomes	Pre-FFCRA Differential Trend	*P*-value
Current Public Coverage	-0.6 (-1.4, 0.2)	0.131
Current Private Coverage	0.5 (-0.2, 1.2)	0.155
Current Uninsurance	-0.0 (-0.3, 0.3)	0.941
Gaps in health coverage	-0.1 (-0.5, 0.3)	0.690
Unmet health care needs	0.1 (-0.1, 0.4)	0.320
Any health care visit	-0.1 (-0.7, 0.5)	0.679
Preventive check-up visit	0.1 (-0.6, 0.8)	0.807

© 2025 Eliason EL et al. *JAMA Health Forum*.

Emergency room visit	-0.2 (-0.9, 0.5)	0.654
Hospital stay	-0.5 (-1.2, 0.1)	0.121
Spent time arranging child's health care	-0.5 (-1.2, 0.1)	0.110
Problems paying health care bills	-0.3 (-0.9, 0.3)	0.335

Notes: The pre-FFCRA period for hospital stay is 2018-2019 and 2017-2019 for all other outcomes.

© 2025 Eliason EL et al. *JAMA Health Forum*.

eTable 4. Event Study Pre-Period Coefficients

The table below presents the pre-FFCRA event study coefficients, calculated as interactions between state continuous eligibility adoption status and each pre-FFCRA year relative to 2019, the last year prior to national continuous eligibility under the FFCRA. Event study pre-period coefficients represent the difference in the outcomes among treated states relative to control group states in each year compared to the reference year, adjusting for state and year fixed effects and control variables presented in the primary difference-in-difference model. The F-statistics represent joint tests of the null hypothesis that all pre-FFCRA yearly coefficients are equal to zero for that outcome. We found no evidence that pre-FFCRA event study coefficients were significantly different from zero for any outcome:

Outcomes	2017 Relative to 2019	*P*-value	2018 Relative to 2019	*P*-value	F-Statistic	*P*-value
Current Public Coverage	1.1 (-0.5, 2.7)	0.178	1.4 (-0.2, 2.9)	0.080	1.70	0.193
Current Private Coverage	-0.8 (-2.2, 0.6)	0.243	-1.7 (-3.3, -0.0)	0.044	2.15	0.127
Current Uninsurance	0.0 (-0.6, 0.7)	0.923	0.0 (-0.6, 0.7)	0.966	0.00	0.995
Gaps in health coverage	0.1 (-0.7, 0.9)	0.725	0.3 (-0.5, 1.1)	0.442	0.30	0.742
Unmet health care needs	-0.3 (-0.8, 0.2)	0.253	0.0 (-0.5, 0.5)	0.941	1.09	0.343
Any health care visit	0.2 (-1.0, 1.4)	0.728	0.4 (-1.2, 2.0)	0.647	0.11	0.893
Preventive check-up visit	-0.2 (-1.7, 1.2)	0.726	0.4 (-1.3, 2.1)	0.623	0.44	0.648
Hospital stay			0.5 (-0.2, 1.2)	0.125	2.44	0.125
Emergency room visit	0.3 (-1.1, 1.7)	0.697	0.6 (-0.7, 1.8)	0.364	0.43	0.656
Spent time arranging child's health care	1.1 (-0.2, 2.5)	0.100	0.4 (-0.6, 1.4)	0.402	1.40	0.255
Problems paying health care bills	0.5 (-0.7, 1.8)	0.373	0.8 (-0.2, 1.7)	0.111	1.34	0.272

Notes: The pre-FFCRA period for hospital stay is 2018-2019 and 2017-2019 for all other outcomes.

© 2025 Eliason EL et al. *JAMA Health Forum*.

eFigure. Unadjusted Trends by State Continuous Eligibility Adoption Status, 2017-2022

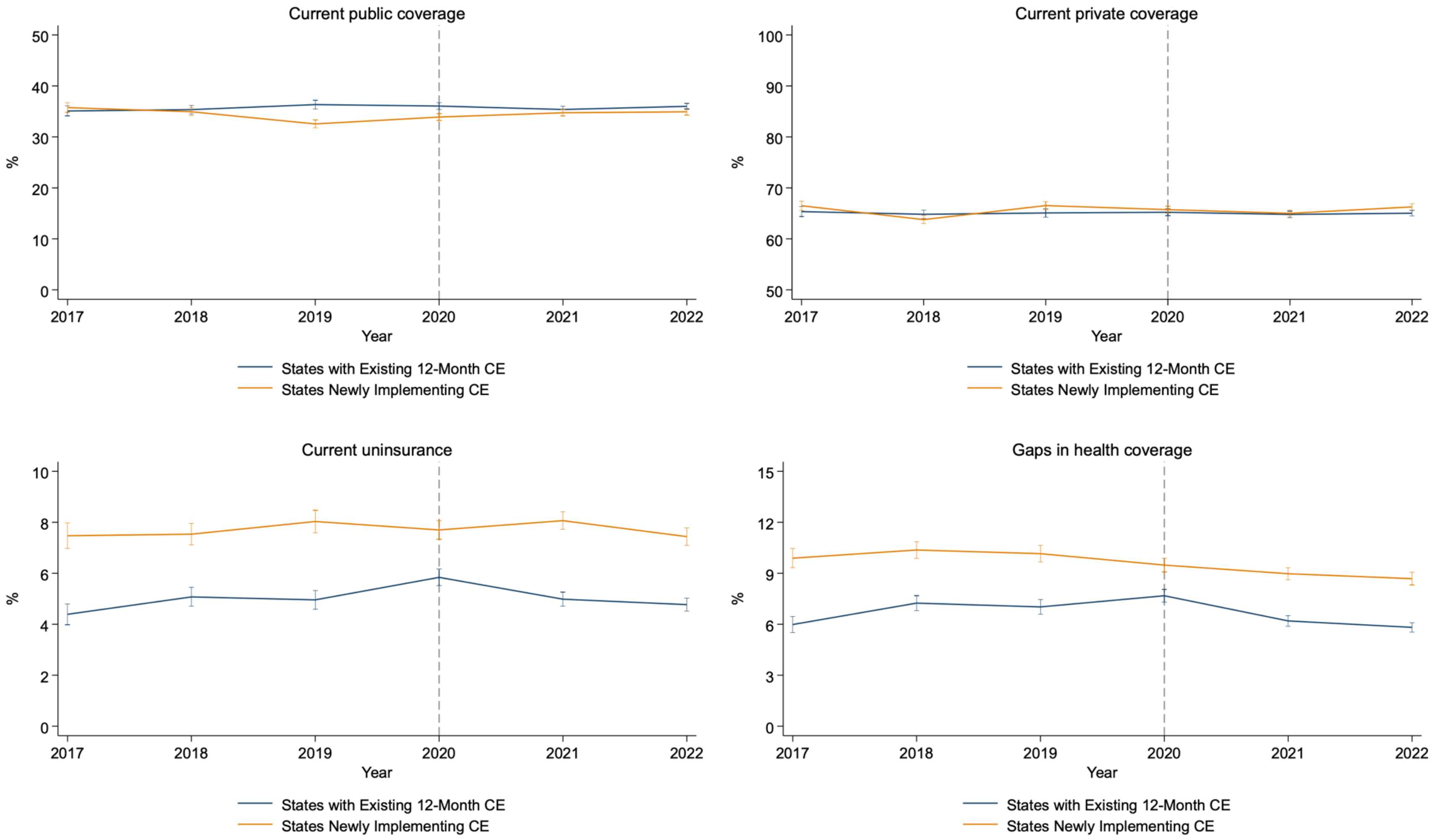

© 2025 Eliason EL et al. *JAMA Health Forum*.

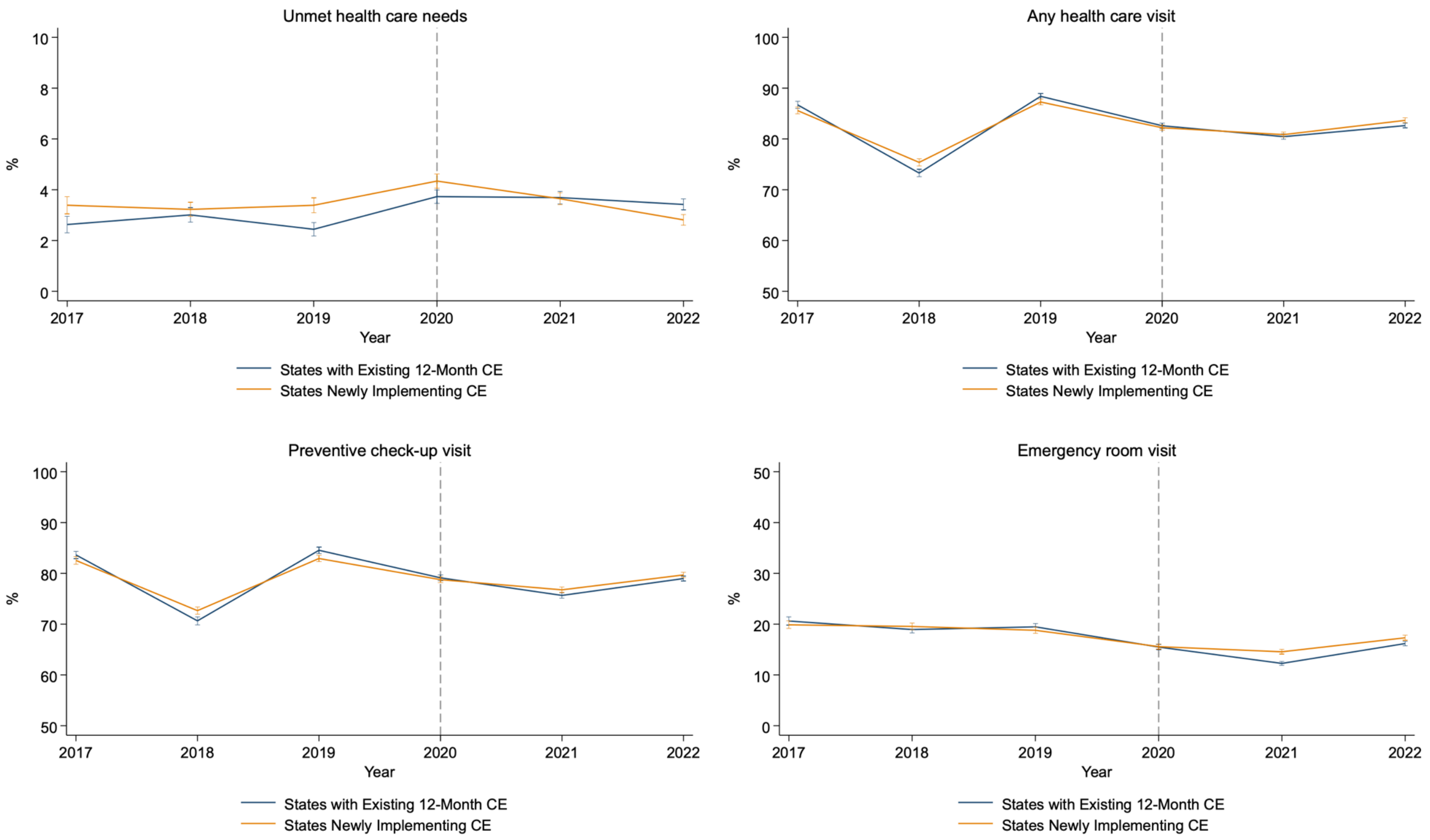

© 2025 Eliason EL et al. *JAMA Health Forum*.

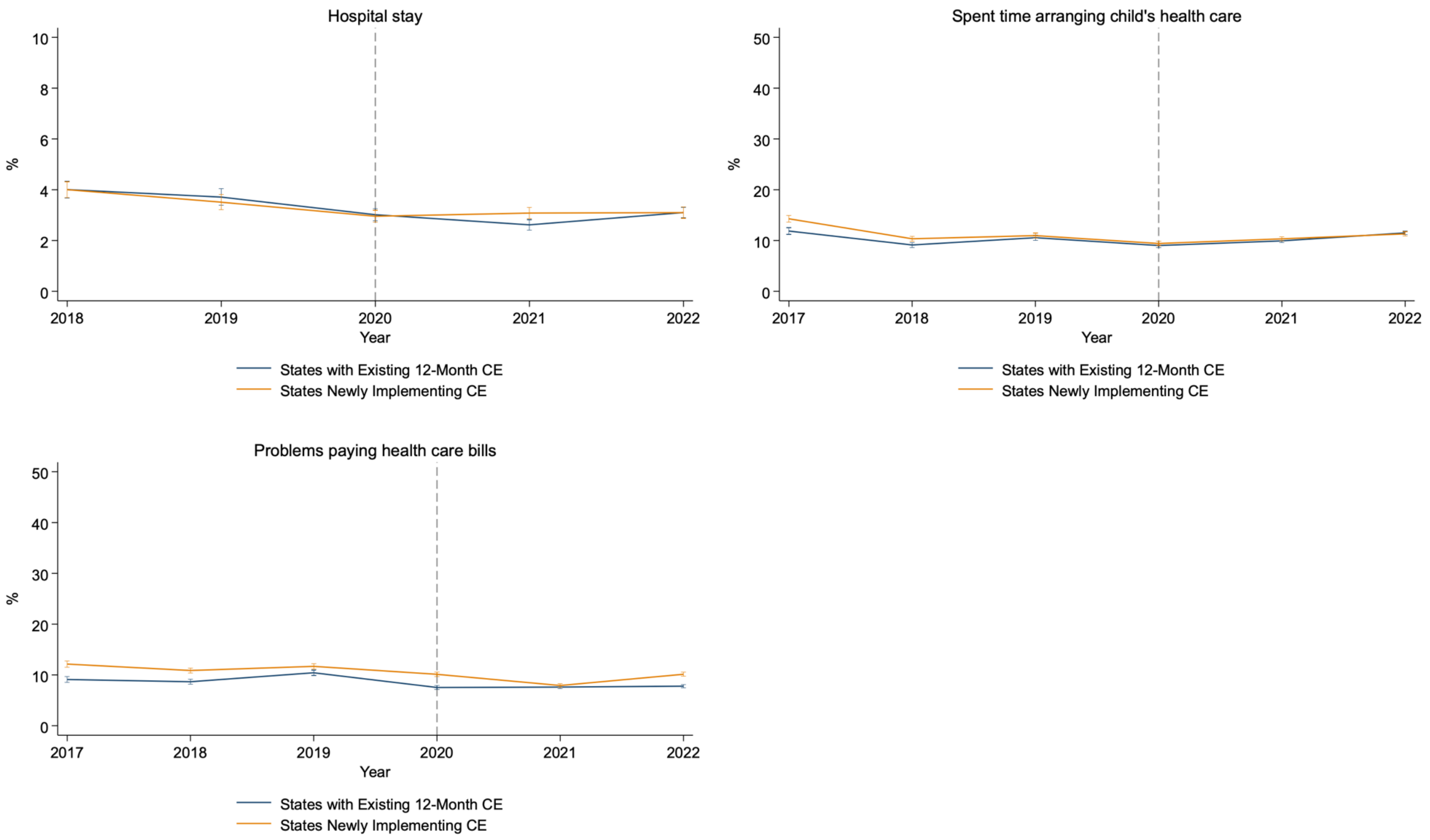

© 2025 Eliason EL et al. *JAMA Health Forum*.

eTable 5. Differential Change in Demographic Characteristics of Publicly Insured Children by State Continuous Eligibility Adoption Status, 2017-2022

As continuous eligibility policies could affect the demographics of publicly insured children, we examined whether FFCRA continuous eligibility was associated with differential changes in child, parent, and household-level demographics by state continuous eligibility adoption status. We found no evidence of significant differential changes in the child, parent, or household demographic characteristics by state continuous eligibility adoption status, suggesting that changes in demographic composition among publicly insured children in states newly implementing continuous eligibility were not driving any differences in children's health care access, use, and barriers to care under the FFCRA.

Outcomes	States with Existing 12-Month CE (24 states)		States Newly Implementing CE (26 states and DC)		Differential Change (95% CI)
	Pre-FFCRA (n=8,700)	During FFCRA (n=19,114)	Pre-FFCRA (n=8,744)	During FFCRA (n=15,689)	
Child age					
Age, years	8.4	8.7	8.1	8.5	9.8 (-29.8, 49.3)
Child gender					
Female	48.6	48.6	48.3	47.6	-0.7 (-4.6, 3.2)
Male	51.4	51.4	51.7	52.4	0.7 (-3.2, 4.6)
Child race and ethnicity					
Non-Hispanic Asian	4.1	3.9	2.9	2.8	0.1 (-1.2, 1.3)
Non-Hispanic Black	20.1	18.8	22.8	20.2	-1.2 (-4.1, 1.8)
Hispanic	36.7	37.1	32.7	34.3	1.0 (-2.4, 4.4)
Non-Hispanic Indigenous	0.6	0.7	0.5	0.5	-0.0 (-0.4, 0.3)
Non-Hispanic Native Hawaiian or Pacific Islander	0.2	0.2	0.3	0.3	0.0 (-0.2, 0.3)
Non-Hispanic White	33.1	33.9	35.3	35.6	-0.3 (-3.2, 2.6)
Non-Hispanic other race	5.2	5.5	5.5	6.1	0.4 (-0.9, 1.7)
Parental age					
Age of mother, years	27.5	27.8	27.0	27.4	15.2 (-33.3, 63.6)
Parental education					

© 2025 Eliason EL et al. *JAMA Health Forum*.

More than high school	50.1	51.3	50.1	48.0	3.3 (-0.5, 7.1)
High school or less	49.9	48.7	49.9	52.0	-3.3 (-7.1, 0.5)
Household nativity					
1st generation household	3.0	4.0	2.9	3.6	-0.3 (-1.7, 1.2)
2nd generation household	30.9	29.9	25.7	25.3	0.3 (-3.3, 4.0)
3rd+ generation	55.4	57.4	59.7	60.9	-0.6 (-4.3, 3.0)
Other	9.5	7.7	11.0	9.4	0.3 (-2.0, 2.6)
Household number of children					
1	24.4	23.5	23.7	24.4	1.6 (-1.3, 4.5)
2	33.8	35.3	32.9	34.3	-0.2 (-3.8, 3.4)
3	24.7	23.6	25.3	24.4	0.2 (-3.4, 3.9)
4 or more	17.1	17.7	18.1	17.0	-1.6 (-4.9, 1.6)
Household language					
English	76.6	73.8	79.1	79.5	3.4 (-0.2, 7.0)
Spanish	18.0	20.5	15.9	16.1	-2.5 (-6.0, 0.9)
Other language	5.4	5.7	5.0	4.4	-0.8 (-2.5, 0.8)

© 2025 Eliason EL et al. *JAMA Health Forum*.

eTable 6. Difference-in-Difference Analysis Omitting 2020 as a Transition Year, 2017-2022
National continuous eligibility under the FFCRA went into effect starting in March 2020. As a result, the majority of months for 2020 were affected by this policy, but January-March 2020 were not. As 2020 was a transition year, we estimated main models omitting the 2020 year. We found results that were similar to main models regarding effect size and statistical significance for current insurance status, coverage gaps, unmet health care needs, health care visits, preventive check-ups, hospital stays, time arranging children's care, and problems paying health care bills. In models omitting 2020 as a transition year, newly implementing continuous eligibility under the FFCRA was additionally associated with a 2.1 percentage point (95% CI: 0.4, 3.7) increase in emergency room visits relative to states with preexisting continuous eligibility.

Outcomes	Unadjusted Difference-in-Differences, (95% CI)	Adjusted Difference-in-Differences, (95% CI)
Current Public Coverage	0.3 (-1.5, 2.0)	0.9 (-0.4, 2.2)
Current Private Coverage	0.3 (-1.0, 1.6)	-0.4 (-1.6, 0.9)
Current Uninsurance	-0.0 (-1.1, 1.0)	0.1 (-0.9, 1.1)
Gaps in health coverage	-0.6 (-2.0, 0.8)	-0.5 (-1.9, 0.9)
Unmet health care needs	-1.0* (-1.8, -0.2)	-1.0* (-1.8, -0.2)
Any health care visit	0.8 (-1.5, 3.1)	0.6 (-1.6, 2.8)
Preventive check-up visit	1.2 (-1.3, 3.7)	1.0 (-1.4, 3.3)
Emergency room visit	2.0* (0.3, 3.7)	2.1* (0.4, 3.7)
Hospital stay	0.3 (-0.3, 0.9)	0.3 (-0.3, 1.0)
Spent time arranging child's health care	-1.2 (-3.1, 0.6)	-1.2 (-3.0, 0.6)
Problems paying health care bills	-0.8 (-2.3, 0.6)	-1.0 (-2.4, 0.5)

© 2025 Eliason EL et al. *JAMA Health Forum*.

eTable 7. Difference-in-Difference Analysis Omitting States with the Highest Numbers of Respondents, 2017-2022
We estimated adjusted difference-in-differences analyses for main models omitting each of the five states with the highest numbers of respondent children one by one. In our study sample, the states with the highest numbers of respondent children were California (7,425 children), Colorado (6,226 children), New York (7,186 children), Oregon (9,472 children), and Wisconsin (5,722 children). In these five models, we found results that were similar to the primary models regarding effect size and statistical significance for the association between newly implementing continuous eligibility under the FFCRA and children's health care access, use, and barriers to care.

Outcomes	Omitting California (*n*= 208,459)	Omitting Colorado (*n*= 209,658)	Omitting New York (*n*= 208,698)	Omitting Oregon (*n*= 206,412)	Omitting Wisconsin (*n*= 210,162)
Current Public Coverage	-0.5 (-2.0, 0.9)	-0.6 (-2.0, 0.7)	-0.6 (-1.9, 0.8)	-0.5 (-1.9, 0.8)	-0.7 (-2.0, 0.6)
Current Private Coverage	0.6 (-0.9, 2.1)	0.6 (-0.8, 1.9)	0.5 (-0.9, 1.9)	0.5 (-0.8, 1.9)	0.6 (-0.8, 1.9)
Current Uninsurance	0.1 (-0.4, 0.6)	-0.3 (-1.0, 0.5)	-0.2 (-1.0, 0.6)	-0.3 (-1.0, 0.5)	-0.3 (-1.0, 0.5)
Gaps in health coverage	-0.3 (-1.0, 0.4)	-0.9 (-2.0, 0.2)	-0.9 (-2.1, 0.2)	-0.9 (-2.0, 0.1)	-0.9 (-2.0, 0.1)
Unmet health care needs	-0.6* (-1.2, -0.0)	-0.7* (-1.3, -0.2)	-0.6* (-1.2, -0.1)	-0.7* (-1.2, -0.2)	-0.7* (-1.2, -0.1)
Any health care visit	-0.6 (-1.8, 0.5)	0.3 (-1.5, 2.2)	0.3 (-1.7, 2.3)	0.3 (-1.5, 2.2)	0.4 (-1.4, 2.2)
Preventive check-up visit	-0.5 (-1.6, 0.6)	0.7 (-1.5, 2.9)	0.6 (-1.7, 3.0)	0.7 (-1.5, 2.8)	0.7 (-1.4, 2.8)
Emergency room visit	0.6 (-0.7, 1.8)	1.4 (-0.4, 3.2)	1.6 (-0.1, 3.4)	1.4 (-0.4, 3.2)	1.4 (-0.4, 3.2)
Hospital stay	0.2 (-0.4, 0.9)	0.2 (-0.4, 0.8)	0.2 (-0.4, 0.8)	0.2 (-0.4, 0.8)	0.3 (-0.3, 0.8)
Spent time arranging child's health care	-0.7 (-2.5, 1.0)	-1.2 (-2.8, 0.5)	-0.8 (-2.4, 0.9)	-1.2 (-2.8, 0.5)	-1.2 (-2.9, 0.5)
Problems paying health care bills	0.0 (-1.2, 1.2)	-0.6 (-2.0, 0.7)	-0.4 (-1.9, 1.0)	-0.7 (-2.0, 0.7)	-0.5 (-1.7, 0.8)

© 2025 Eliason EL et al. *JAMA Health Forum*.

eTable 8. Difference-in-Difference Analysis with Multiply Imputed Federal Poverty Level, 2017-2022

The Census Bureau addresses small amounts of missingness in the NSCH data using a hot-deck imputation approach for child gender, race, and ethnicity and sequential regression imputation methods for parental education (U.S. Census Bureau, 2024b; U.S. Census Bureau, 2024c). For all other missingness among the covariates, we excluded observations with missing data. Approximately 15% of the NSCH sample has missing data for information to calculate the federal poverty level (FPL). As a result, the Census Bureau uses a multiple imputation approach to calculate FPL measures, resulting in six imputed values in the NSCH data (U.S. Census Bureau, 2024b). As a sensitivity analysis, we included FPL as an additional covariate, accounting for the multiple imputation framework for the FPL variable using the multiple imputation command in Stata. Using this approach, we found results that were consistent with main models regarding effect size and statistical significance for the association between state continuous eligibility adoption status and children's health care access, use, and barriers to care during the FFCRA.

Outcomes	Adjusted Difference-in-Differences, (95% CI)	*P*-value
Current Public Coverage	-0.4 (-1.4, 0.7)	0.495
Current Private Coverage	0.4 (-1.0, 1.7)	0.580
Current Uninsurance	-0.3 (-1.0, 0.5)	0.495
Gaps in health coverage	-0.9 (-1.9, 0.2)	0.108
Unmet health care needs	-0.7 (-1.2, -0.2)	0.011
Any health care visit	0.3 (-1.5, 2.1)	0.727
Preventive check-up visit	0.7 (-1.5, 2.8)	0.531
Emergency room visit	1.4 (-0.3, 3.1)	0.109
Hospital stay	0.2 (-0.3, 0.8)	0.408
Spent time arranging child's health care	-1.2 (-2.8, 0.4)	0.151
Problems paying health care bills	-0.6 (-1.9, 0.6)	0.318

eReferences

Health Resources and Services Administration (HRSA) Maternal and Child Health Bureau (MCHB) (2024). The National Survey of Children's Health. https://www.childhealthdata.org/learn-about-the-nsch/NSCH

U.S. Census Bureau (2024a). 2020 National Survey of Children's Health: Methodology Report. April 10, 2024. https://www2.census.gov/programs-surveys/nsch/technical-documentation/methodology/2020-NSCH-Methodology-Report.pdf

U.S. Census Bureau (2024b). Data Users Frequently Asked Questions (FAQs). National Survey of Children's Health. August 20, 2024. https://www2.census.gov/programs-surveys/nsch/technical-documentation/methodology/2022-NSCH-FAQs.pdf

U.S. Census Bureau (2024c). Analysis with Multiply Imputed Data. National Survey of Children's Health. April 10, 2024. https://www2.census.gov/programs-surveys/nsch/technical-documentation/methodology/NSCH-Analysis-with-Imputed-Data-Guide.pdf

© 2025 Eliason EL et al. *JAMA Health Forum*.
