# Supplementary material for: Children’s Continuous Medicaid Eligibility During COVID-19 and Health Care Access, Use, and Barriers to Care
Source: JAMA Health Forum. 2025 Jun 13;6(6):e251376. doi: 10.1001/jamahealthforum.2025.1376 (PMC12166482; doi:10.1001/jamahealthforum.2025.1376)
Supplement: Supplement 2. — Data Sharing Statement [file jamahealthforum-e251376-s002.pdf]

Data Sharing Statement

Eliason. Children's Continuous Medicaid Eligibility During COVID-19 and Health Care Access, Use, and Barriers to Care. *JAMA Health Forum*. Published June 13, 2025. doi:10.1001/jamahealthforum.2025.1376

Data
Data available: Yes
Data types: Other (please specify)
Additional Information: Data is from a publicly available source, the National Survey of Children's Health
How to access data: https://www.census.gov/programs-surveys/nsch/data/datasets.html
When available: With publication

Supporting Documents
Document types: None

Additional Information
Who can access the data: Anyone requesting the data
Types of analyses: For any purpose
Mechanisms of data availability: Data is from a publicly available source, the National Survey of Children's Health
Any additional restrictions: None
